# Promoter swapping of truncated *PDGFRB* drives Ph-like acute lymphoblastic leukemia

**DOI:** 10.1038/s41698-023-00485-7

**Published:** 2023-12-09

**Authors:** Bunpei Miyazaki, Toshihide Ueno, Masanaka Sugiyama, Shinya Kojima, Ayumu Arakawa, Kayoko Tao, Kazuki Tanimura, Kouya Shiraishi, Shigehiro Yagishita, Shinji Kohsaka, Mamoru Kato, Nobutaka Kiyokawa, Yasushi Goto, Yasushi Yatabe, Akinobu Hamada, Hiroyuki Mano, Chitose Ogawa, Yosuke Tanaka

**Affiliations:** 1 Department of Pediatric Oncology, National Cancer Center Hospital, Tokyo, 104-0045 Japan; 2 Division of Cellular Signaling, National Cancer Center Research Institute, Tokyo, 104-0045 Japan; 3 Department of Clinical Genomics, National Cancer Center Research Institute, Tokyo, 104-0045 Japan; 4 Division of Molecular Pharmacology, National Cancer Center Research Institute, Tokyo, 104-0045 Japan; 5 Division of Bioinformatics, National Cancer Center Research Institute, Tokyo, 104-0045 Japan; 6 Department of Pediatric Hematology and Oncology Research, National Research Institute for Child Health and Development, Tokyo, 157-0074 Japan; 7 Department of Thoracic Oncology, National Cancer Center Hospital, Tokyo, 104-0045 Japan; 8 Department of Diagnostic Pathology, National Cancer Center Hospital, Tokyo, 104-0045 Japan

**Keywords:** Acute lymphocytic leukaemia, Cancer

## Abstract

Philadelphia chromosome (Ph)-like acute lymphoblastic leukemia (ALL) is a subset of ALL that demonstrated a high treatment failure rate. One of the hallmarks of Ph-like ALL is PDGFRB gene fusion, with fusion partner proteins often harboring dimerization domains and enhancing the kinase activity of PDGFRB. We determined a novel oncogenic PDGFRB fusion gene, NRIP1::PDGFRB, from a pediatric patient with ALL, encoding a protein with the carboxy-terminal kinase domain of PDGFRB, without the partner peptide. We confirmed the oncogenic potential of NRIP1::PDGFRB in vitro and the efficacy of all ABL1-specific inhibitor generations, including imatinib, dasatinib, nilotinib, and ponatinib, in suppressing this potential. PDGFRB activation mechanism may include juxtamembrane domain truncation in the predicted peptide. In conclusion, we determined a novel fusion gene pattern in Ph-like ALL.

## Introduction

The development of a risk classification strategy based on molecular subtyping has significantly improved the prognosis of childhood acute lymphoblastic leukemia (ALL) in recent decades^1^. Risk classification contributed to adapting appropriate treatment options, such as intensified treatment and molecular targeting agents for patients at adverse risk, or treatment with reduced intensity for patients at favorable risk. Philadelphia chromosome (Ph)-like ALL, demonstrates a gene expression profile similar to *BCR*::*ABL1*-positive ALL^2^ and accounts for 15%–30% of B-cell lineage ALL (B-ALL) in children and adults^3^. Ph-like ALL is associated with high rates of treatment resistance and relapse^4^. The 5-year event-free survival rates are ~60% and 80% in Ph-like ALL and other childhood ALL subtypes, respectively^5^. Ph-like ALL often carries oncogenic fusions of tyrosine kinases, including *PDGFRB* fusions. Most *PDGFRB* fusions include amino (N)-terminal partner protein with a dimerization motif, such as EBF1, and carboxy (C)-terminal kinase domain of PDGFRB^3^. The dimerization motif facilitates homodimer formation of the kinase domain, causing autophosphorylation^6^. Herein, we report a novel truncated form of PDGFRB without a partner protein in B-ALL and confirm its oncogenicity and sensitivity to tyrosine kinase inhibitors (TKIs).

## Results

**Fig. 1:**
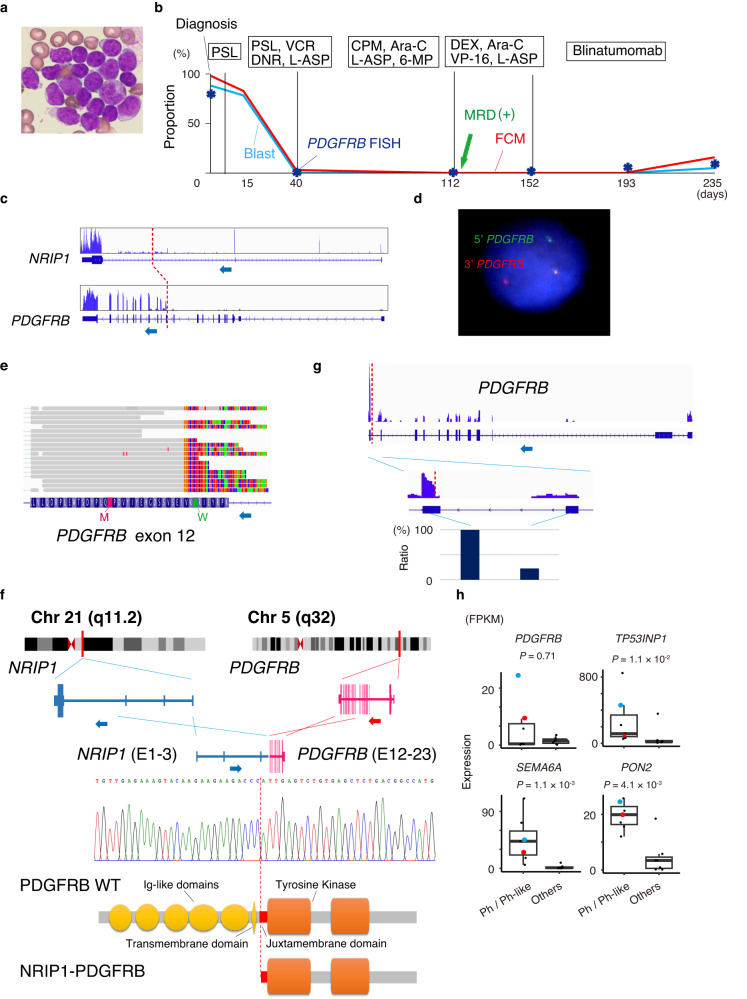
Identification of the *NRIP1*::*PDGFRB* fusion gene in a Ph-like ALL patient. **a** Representative Giemsa staining of leukemic cells. **b** Clinical timeline of patient’s treatment history from diagnosis; treatments at different time points are shown along the top. The blue and red lines indicate the ratio of blasts and tumor cells detected by flow cytometry (FCM). The asterisk indicates the ratio of *PDGFRB* FISH-positive cells. PSL prednisolone, VCR vincristine, DNR daunorubicin, L-ASP L-asparaginase, 6-MP mercaptopurine, CPM cyclophosphamide, Ara-C cytarabine, DEX dexamethasone, VP-16 etoposide. Intrathecal chemotherapy was administered throughout each treatment phase. Minimal residual disease (MRD) positivity was detected on Day 112. **c** Reads of *NRIP1*::*PDGFRB* fusion in each genomic locus; dashed line indicates the genomic breakpoint. **d** *PDGFRB* break-apart FISH analysis is depicted; green and red dots indicate 5' and 3' ends of *PDGFRB* DNA probe. **e** Chimeric reads of *NRIP1*::*PDGFRB* fusion in exon12 of *PDGFRB;* colored portions of the reads indicate mismatched bases. M methionine, W tryptophan. **f** Schematic representation of *NRIP1*::*PDGFRB* fusion. **g** Reads in *PDGFRB* locus; dashed line indicates the genomic breakpoint, and bottom panel shows the ratio of reads between exons before and after the breakpoint. **h** Expression levels of *PDGFRB* and the representative genes downstream of activated tyrosine kinases in ALL in Ph-like and Ph ALL groups (left, *n* = 7) and ALLs of other subtypes (right, *n* = 7); red and blue dots indicate cases with *NRIP1*::*PDGFRB* and *EBF1*::*PDGFRB* fusions. The box plots show medians (lines), interquartile ranges (IQRs; boxes), and ± 1.5 × IQRs (whiskers).

A 4-year-old patient visited the National Cancer Center Hospital in Japan with complaints of fever, malaise, and purpura. Peripheral blood examination revealed 8.4 × 10^10^/L white blood cells with 81% blasts (Fig. 1a), 5.6 g/dL hemoglobin, and 1.6 × 10^10^/L platelets. Bone marrow aspiration revealed 90% of myeloperoxidase-negative blasts. Cell surface marker profiling with flow cytometry demonstrated that the blasts were positive for CD19, CD10, CD22, cyCD79a, cy-μ chain, CD27, CD44, and CD66c and negative for T-cell and myeloid markers. The patient was diagnosed with pre-B-ALL based on these results. Figure 1b shows her clinical course. The patient was treated according to the Japanese Pediatric Leukemia/Lymphoma Study Group B-19 protocol and refractory to the early phase of induction chemotherapy, including prednisolone. Complete remission was eventually achieved after the entire induction phase, but minimal residual disease was detected after the early consolidation phase. The patient was refractory to the salvage chemotherapy with blinatumomab, a bispecific antibody against CD3 and CD19 that induces anti-tumor T-cell responses. The patient was scheduled for chimeric antigen receptor T-cell therapy followed by allogeneic hematopoietic stem cell transplantation. The patient’s parents provided informed consent for genetic analyses during induction chemotherapy.

RNA sequencing (RNA-seq) of leukemia cells revealed the presence of a novel *NRIP1*::*PDGFRB* fusion gene, where an untranslated region of *NRIP1* intron 3 was connected to exons 12–23 of *PDGFRB* (Fig. 1c). Cytogenetic analysis with fluorescence in situ hybridization (FISH) revealed a split signal of *PDGFRB* in 88/100 leukemic cells analyzed (Fig. 1d). Whole exome sequencing of leukemic blasts revealed no other genetic abnormalities. Interestingly, the fusion gene breakpoint resides in the middle of exon 12 of *PDGFRB*, followed by methionine for translation initiation (Fig. 1e). Thus, the protein resulting from *NRIP1*::*PDGFRB* has an amino acid sequence for C-terminus of *PDGFRB*, with a conserved tyrosine kinase domain, while it lacks N-terminal extracellular and juxtamembrane (JM) domains (Fig. 1f). The coding sequence of *NRIP1* is excluded from the fusion transcript; thus, promoter swapping was considered a potential mechanism for the increased expression of truncated *PDGFRB* caused by this translocation. The increase in the expression levels of *PDGFRB* was detected between exons before and after the breakpoint, indicating the result of promoter swapping in the translocated allele (Fig. 1g). Considering the expression levels of representative genes downstream of activated tyrosine kinases in ALL^7^ from the data of our previous study^8^, our case belonged to Ph-like/Ph-positive group (Fig. 1h). Additionally, *PDGFRB* expression levels increased in *PDGFRB* rearranged cases, including this one.

**Fig. 2:**
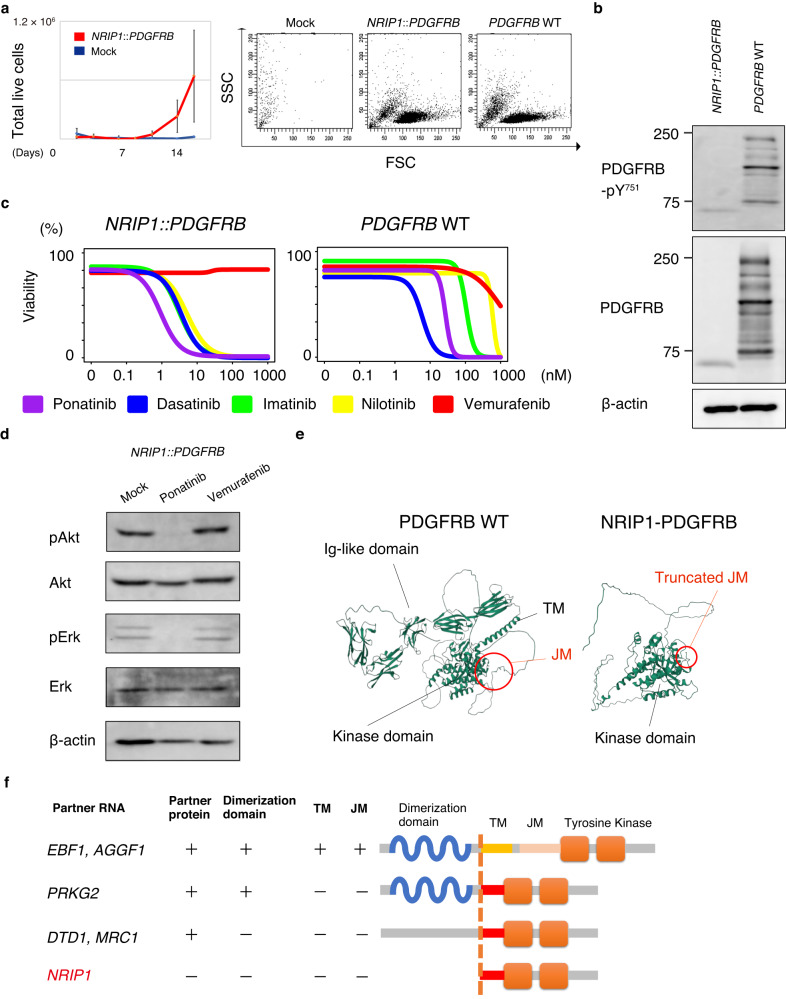
Oncogenicity of the *NRIP1*::*PDGFRB* fusion gene. **a** Left panel shows Ba/F3 outgrowths transduced with *NRIP1*::*PDGFRB* compared to Ba/F3 cells transduced with a mock vector upon IL-3 withdrawal; right panel shows flow cytometry images of Ba/F3 cells transduced with a mock vector, including *NRIP1*::*PDGFRB* and *PDGFRB* WT; transformed clones were detected only in Ba/F3 cells transduced with *NRIP1*::*PDGFRB* and *PDGFRB* WT vectors; FSC forward scatter, SSC side scatter. **b** Western blot analyses of Ba/F3 cells transduced with *NRIP1*::*PDGFRB* or *PDGFRB* WT vectors; truncated form of PDGFRβ and phosphorylated PDGFRβ were shown on the left. **c** Ba/F3 cell sensitivities transduced with *NRIP1*::*PDGFRB* or *PDGFRB* WT vectors to imatinib, dasatinib, nilotinib, ponatinib, and vemurafenib; data are presented as the average of three independent experiments; vertical axis indicates viability as calculated by cell number. **d** Western blot analysis of Ba/F3 cells, transduced with *NRIP1*::*PDGFRB*, treated with ponatinib (1 nM) and vemurafenib (1 nM); after 24 h of tyrosine kinase inhibitor exposure, lysates were prepared and immunoblotted. **e** Structures of WT PDGFRB and NRIP1::PDGFRB predicted with AlphaFold2. TM transmembrane domain, JM juxtamembrane domain. **f** Schematic representation of *PDGFRB* fusion pattern; TM transmembrane domain, JM juxtamembrane domain.

We stably transduced a murine pro-B-cell line, Ba/F3, with *NRIP1*::*PDGFRB* to validate the oncogenic potential of *NRIP1*::*PDGFRB*. Wild-type (WT) *PDGFRB* cDNA was also transduced as a positive control. Ba/F3 cells expressing *NRIP1*::*PDGFRB* and WT *PDGFRB* cDNA survived upon IL-3 withdrawal for 1 week (Fig. 2a). We detected *NRIP1*::*PDGFRB-generated* truncated form of *PDGFRB* and its phosphorylation (Fig. 2b), as well as the excessive phosphorylation of downstream targets of *NRIP1*::*PDGFRB* (Fig. 2d). Next, we incubated these cells with different concentrations of known ABL1 TKIs and one BRAF kinase inhibitor (vemurafenib). As demonstrated in Fig. 2c, Ba/F3 cells that express *NRIP1*::*PDGFRB* or WT *PDGFRB* were sensitive to all ABL1 TKI generations (imatinib, dasatinib, nilotinib, and ponatinib), with a trend toward a lower IC50 in cells expressing *NRIP1*::*PDGFRB* than WT *PDGFRB*. Reduced phosphorylation of downstream targets of *NRIP1*::*PDGFRB* was achieved by ABL1 TKI administration, but not by other TKIs, supporting the results of the drug sensitivity assay (Fig. 2d).

## Discussion

Patients with Ph-like ALL frequently show tyrosine kinase fusions, and *EBF1* is a major fusion partner of *PDGFRB*, found in 73% of fusions^3^. To date, almost all fusion partners carry dimerization motifs, such as the coiled-coil domain. Fusion to the protein with dimerization motifs results in *PDGFRB* kinase domain homodimerization, causing kinase autophosphorylation and activation. Such a response potentiates RAS/MAPK and PI3K pathways and promotes cell proliferation^6^.

In contrast, the encoded protein by *NRIP1*::*PDGFRB* lacks a partner protein with a dimerization domain, although we confirmed its growth-inducing ability through excessive autophosphorylation. Although rare, hematological malignancies demonstrated *PDGFRB* fusions without a dimerization protein^9^. The partner proteins of *PDGFRB* in the fusion protein encoded by *DTD1*::*PDGFRB*, *MRC1*::*PDGFRB* fusions^10,11^, and *G3BP1*::*PDGFRB* demonstrated no dimerization domains while the oncogenic ability has been experimentally confirmed^12^.

The characteristics of these fusion protein types are truncated JM domain, detected in our case. JM domain in tyrosine kinase receptors has been reported as an autoinhibitory domain that suppresses kinase activity through conformational proximity^13^. Additionally, some tyrosine kinase families, other than *PDGFRB*, are activated by JM dysfunction. FLT3 and KIT have crystal structures similar to *PDGFRB*. An internal tandem duplication (ITD) of JM domain in FLT3 causes a structural alteration of JM domain, resulting in constitutive activation of its enzymatic function and cell proliferation^14^. FLT3-ITD alterations occur in acute myeloid leukemia (AML), accounting for ~30% of AML cases^15^. Mutations in JM domain of KIT in gastrointestinal stromal tumors (GIST) demonstrated a similar activation mechanism^16^. TKIs are effective and clinically used for FLT3-ITD-positive AML and KIT rearranged GISTs^17,18^. *FIP1L1::PDGFRA* fusion, one of the major oncogenic fusion genes of myeloproliferative disorders, is another example of JM dysfunction. The translated protein contains truncated JM and kinase domains of *PDGFRA*^19^. Similarly, proliferative ability^20^ and response to TKI^21^ have been demonstrated.

Importantly, Stover et al. revealed an increase in enzymatic ability with the absence of tryptophan-566 (W566) in JM region of *PDGFRB*^20^, and Chen et al. reported the crucial role of W566 in maintaining JM domain assembly^22^. The protein encoded by *NRIP1*::*PDGFRB*, in our case, lacks JM domain (Fig. 2e); the fusion transcript excluded the sequence encoding W566 (Fig. 1e). A recent study reported a novel *PDGFRB* fusion gene, *CD74*::*PDGFRB*, in Ph-like ALL in addition to the known patterns of *PDGFRB* fusions (Fig. 2f)^23^. The sequence encoding W566 was conserved in the transcript, but *PDGFRB* translation starts from the same translation start site as *NRIP1*::*PDGFRB*, causing the same form of JM truncated *PDGFRB* protein. Specifically, they experimentally revealed that the truncated *PDGFRB* without W566 harbors a stronger kinase activity than truncated *PDGFRB* retaining W566. Additionally, they revealed that *CD74*::*PDGFRB* did not dimerize as strongly as *EBF1*::*PDGFRB*, a representative *PDGFRB* fusion gene with partner protein harboring dimerization domain. *PDGFRB* protein with truncated JM results in excessive downstream phosphorylation as shown in our case, but dimerization may not be necessary for *PDGFRB* autophosphorylation in JM dysregulated cases. Altogether, truncated JM is a novel oncogenic form of *PDGFRB* aberration in Ph-like ALL.

The 5-year event-free survival of Ph-like ALL with *PDGFRB* rearrangement was 50%^24^. Accumulating reports indicated the efficacy of TKIs against Ph-like ALL, including those with *PDGFRB* rearrangement^25–27^, although they remained prospectively not validated. Currently, an ongoing prospective trial aims to confirm the efficacy of dasatinib in patients with Ph-like ALL with specific fusions (Children’s Oncology Group’s AALL1131, NCT02883049). We and others^23^ confirmed the proliferative capacity and response to TKIs in JM dysregulated *PDGFRB*; thus, our data will be beneficial for future patient selection. In conclusion, our study identified a novel truncated *PDGFRB* fusion in Ph-like ALL without fusion partner peptides which can be targeted by TKIs.

## Methods

### Sample

We used a bone marrow aspiration specimen for RNA sequencing (RNA-seq). The patient’s parents signed a written informed consent for genetic analyses and publication of the case report. The National Cancer Center Research Ethics Review Board approved this study (2015-059). We followed the ethical principles of the Declaration of Helsinki.

### RNA sequencing

We extracted total RNA from the bone marrow sample and prepared and subjected RNA-seq libraries to next-generation sequencing as previously described^28^. We used Arriba to detect gene fusion^29^.

### Primers for NRIP1::PDGFRB fusion

We identified *NRIP1*::*PDGFRB* fusion transcript by cDNA PCR from the patient sample using the following primer sets:

*NRIP1* forward: TTGGATTGTGAGCTATTTCAGAAC

*PDGFRB* reverse: AGGGTTTGGGGCACAACACGTCAG

### Cell culture

The wild-type *PDGFRB* cDNA and *NRIP1*::*PDGFRB* cDNA coding regions were inserted into the pMXS plasmid. Ba/F3 cells were infected with the generated retroviruses from each plasmid.

### Drug sensitivity assay

Cells were seeded into 96-well plates at a 100 μL volume. After overnight incubation, cells were treated with each drug, including imatinib (Selleck), dasatinib (Selleck), nilotinib (Selleck), ponatinib (Selleck), and vemurafenib (Selleck), at doses ranging from 0.1 nM to 1 μM, incubated for 72 h. Subsequently, 10 μL of PrestoBlue (Thermo Fisher Scientific) was added to the plates, and the fluorescence was measured after 3 h of incubation.

### Clinical sequence data

Sequencing data of Japan Adult Leukemia Study Group (JALSG) B-ALL clinical samples were obtained from the Japanese Genotype–Phenotype Archive (accession JGAS00000000047)^8^, which is hosted by the DNA Databank of Japan.

### Western blot

Standard protocols were used for protein detection by immunoblot analysis, using primary antibodies PDGFRβ (#3169, 1:1000 dilution), phospho-PDGFRβ (Tyr751) (#3161, 1:1000 dilution), Akt (#4691, 1:1000 dilution), phospho-Akt (Ser473) (#4060, 1:1000 dilution), Erk1/2 (#4695, 1:1000 dilution), phospho-Erk1/2 (#4370, 1:1000 dilution), and β-Actin (#4970, 1:1000 dilution) purchased from Cell Signaling Technology. Uncropped immunoblots blots of each Figure are included in Supplementary Fig. 1.

### Reporting summary

Further information on research design is available in the Nature Research Reporting Summary linked to this article.

### Supplementary information

Supplementary Information

Reporting Summary

## Data Availability

Sequencing data is deposited at Gene Expression Omnibus (GEO) under the accession number GSE242858.
