# Supplementary material for: Promoter swapping of truncated PDGFRB drives Ph-like acute lymphoblastic leukemia
Source: NPJ Precis Oncol. 2023 Dec 9;7:132. doi: 10.1038/s41698-023-00485-7 (PMC10710492; doi:10.1038/s41698-023-00485-7)
Supplement: Supplementary file 1 — Supplementary Information [file 41698_2023_485_MOESM1_ESM.pdf]

Supplementary Figures and Legends

Figure 2b

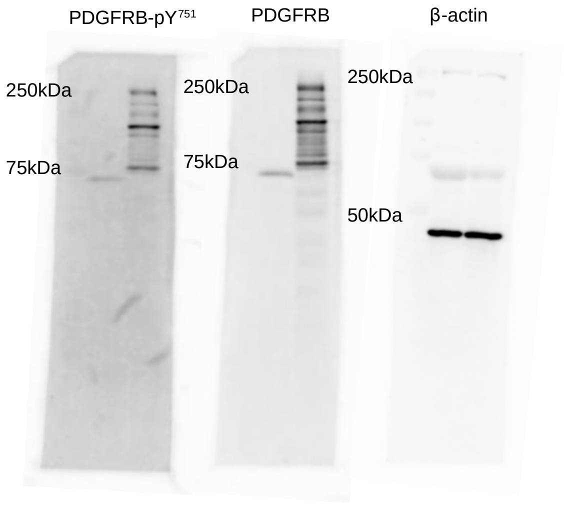

Figure 2d

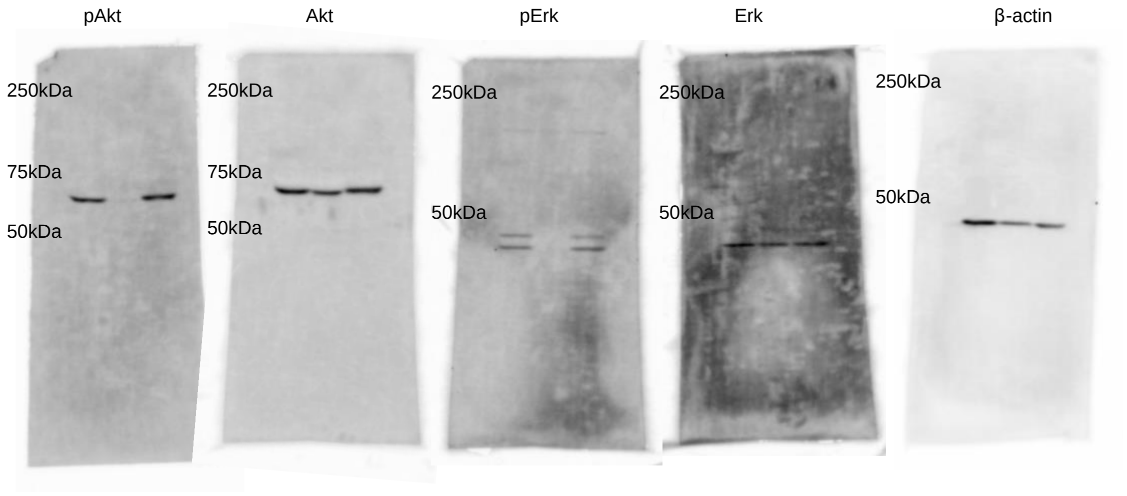

Supplementary Figure 1

Uncropped immunoblots blots

Uncropped immunoblots blots of each Figure are shown.
